# Adaptation of a difficult-to-manage asthma programme for implementation in the Dutch context: a modified e-Delphi

**DOI:** 10.1038/npjpcrm.2016.86

**Published:** 2017-02-09

**Authors:** Persijn J Honkoop, Hilary Pinnock, Regien M M Kievits-Smeets, Peter J Sterk, P N Richard Dekhuijzen, Johannes C C M in ’t Veen

**Affiliations:** 1 Department of Quality of Care, Leiden University Medical Center, Leiden, The Netherlands; 2 Asthma UK Centre for Applied Research, Usher Institute of Population Health Sciences and Informatics, University of Edinburgh, Edinburgh, UK; 3 COPD Astma Huisartsen Advies Groep (CAHAG), Vught, The Netherlands; 4 Department of Respiratory Medicine, Academic Medical Center, Amsterdam, The Netherlands; 5 Department of Pulmonary Medicine, Radboud University Medical Center, Nijmegen, The Netherlands; 6 Department of Pulmonology, STZ Centre of Excellence for Asthma and COPD, Franciscus Gasthuis and Vlietland, Rotterdam, The Netherlands

## Abstract

Patients with difficult-to-manage asthma represent a heterogeneous subgroup of asthma patients who require extensive assessment and tailored management. The International Primary Care Respiratory Group approach emphasises the importance of differentiating patients with asthma that is difficult to manage from those with severe disease. Local adaptation of this approach, however, is required to ensure an appropriate strategy for implementation in the Dutch context. We used a modified three-round e-Delphi approach to assess the opinion of all relevant stakeholders (general practitioners, pulmonologists, practice nurses, pulmonary nurses and people with asthma). In the first round, the participants were asked to provide potentially relevant items for a difficult-to-manage asthma programme, which resulted in 67 items. In the second round, we asked participants to rate the relevance of specific items on a seven-point Likert scale, and 46 items were selected as relevant. In the third round, the selected items were categorised and items were ranked within the categories according to relevance. Finally, we created the alphabet acronym for the categories ‘the A–I of difficult-to-manage asthma’ to resonate with an established Dutch ‘A–E acronym for determining asthma control’. This should facilitate implementation of this programme within the existing structure of educational material on asthma and chronic obstructive pulmonary disease (COPD) in primary care, with potential for improving management of difficult-to-manage asthma. Other countries could use a similar approach to create a locally adapted version of such a programme.

## Introduction

Asthma is a common respiratory condition, accounting for serious morbidity for patients and an important negative impact on society and economy owing to high costs of medication, hospitalisation and loss of productivity.^1–3^ Even now, 60% of the asthmatics are poorly controlled, which is related to a significant reduction in quality of life.^4,5^

Mortality remains high despite the availability of effective therapies.^6^ Recently, major preventable factors were found in 67% of asthma deaths, and only a small portion of patients had such severe asthma that mortality could not have been prevented.^7^ Therefore, it is of vital importance to differentiate between patients with truly severe asthma (estimated as 3.6% of people with asthma in the Netherlands) from persons with difficult-to-manage asthma (estimated as 17.4% of people with asthma).^8^ Difficult-to-manage asthma is asthma that either the person affected or the clinician finds difficult to manage, which may or may not be driven by the severity of the disease.^9^ It requires identification and management of treatable traits,^10^ predominantly beyond pharmacotherapy.^11^ By doing so, the potentially life-saving but thus far very costly new types of treatment can be reserved for the patients with truly severe asthma.^12,13^ International guidelines define severe asthma as asthma that requires treatment with high-dose inhaled corticosteroids plus a second controller and/or systemic corticosteroids to prevent it from becoming ‘uncontrolled’, or that remains ‘uncontrolled’ despite this therapy, when supervised by a specialised multidisciplinary team for at least 3 months.^4,14,15^

Within this definition, a systematic approach to reviewing all potentially preventable and treatable factors before stepping up treatment and/or deciding asthma is severe is not explicitly specified, though guidelines do advocate reviewing preventable factors. In addition, assessment of difficult-to-manage asthma in primary care might be hampered by a lack of knowledge about all potentially preventable or treatable factors.

Therefore, Ryan *et al.*^16^ have developed the SIMPLES approach to the primary care management of ‘difficult-to-manage’ asthma, adopted by the IPCRG (International Primary Care Respiratory Group) in an international ‘Train the Trainer’ initiative.^17^ The approach identifies patients who are currently not controlled, systematically addresses potentially preventable or treatable factors and distinguishes those with difficult-to-manage asthma from those with severe asthma, enabling primary care physicians to guide treatment with proper allocation to primary care or hospital care management. This approach originated in the UK, and before it can be successfully implemented in another country, it is important to address potential barriers, such as differences in healthcare systems and language issues.^18–22^ Furthermore, several educational programmes regarding the management of asthma already exist in primary care in the Netherlands.^23^ Therefore we aimed to design a programme for difficult-to-manage asthma, analogous to SIMPLES, which is tailored to the context in the Netherlands.

## Results

### Participants

**Figure 1:**
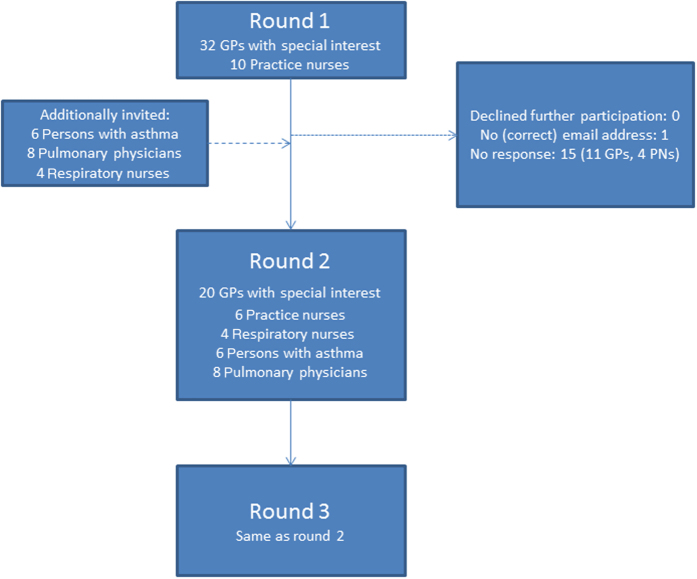
Flowchart of study participants. GP, general practitioner; PN, practice nurse.

Figure 1 presents a flowchart of the participants of the three rounds (see the Materials and Methods' section for a description of the three rounds). All the first round respondents were invited to participate in the final two rounds. In addition, several pulmonary physicians, respiratory nurses and patients were added for rounds 2 and 3. Characteristics of the members of the final two Delphi rounds are presented in Table 1.

### Items generated and ranked for clinical relevance

The first Delphi round resulted in a list of 67 items, presented in Table 2. A total of 46 items met the second-round inclusion criterion of 80% of participants scoring a 5, 6 or 7 (above-average relevance), also presented in Table 2 under the heading selected yes/no. For the third round, we categorised the selected items in the following categories:

- Background
- Diagnosis
- Monitoring
- Exacerbation
- Types of medication
- Use of medication
- Smoking
- Other irritants
- Lifestyle
- Education and self-management
- Patient Profile
- Individual care plan

The ranking of items within each of these categories is given in Table 3.

### Developing an acronym

After analysis of the categories and the ranked items within the categories, we decided to use the letters A–I of the alphabet, resulting in ‘The A–I of difficult to manage asthma’ (see Table 4). Our main reasons were as follows:

- It is an easy-to-remember and clear acronym.
- In some parts in the Netherlands, a short version already exists, which is used to determine the cause of insufficient asthma control (The A–E of asthma).^24^ By extending an existing acronym, we hoped to facilitate implementation.
- All the relevant categories and items could be addressed within the acronym.

### Subgroup analyses

The results of the subgroup analyses comparing responses of patients and professionals and between primary care and hospital care are given in an online supplement (Supplementary Tables 5a and 5b). There was no significant difference between patients and professionals in the number of items they prioritised (i.e., scored ⩾5), respectively, 82% vs. 80%, odds ratio=0.89 (95% confidence interval: 0.68 to 1.17), though primary care professionals prioritised significantly fewer items than hospital specialists (respectively, 77% vs. 85%, odds ratio=1.69, 95% confidence interval: 1.34 to 2.12). Hospital care professionals assigned a higher score to a wide variety of items, both clinical, such as role of blood eosinophils and phenotypes, and behavioural parameters, such as adherence, involving patients’ peers in management, attention for physical activity and weight.

In another subgroup analysis, we compared the prioritisation of items by primary and hospital care professionals and results are presented in Supplementary Table 6 of the online supplement. Views differed mostly in the headings Diagnosis and Education and self-management.

## Discussion

### Main findings

We devised a new educational programme for promoting structured management of difficult-to-manage asthma in Dutch primary care. Our starting point was the SIMPLES programme, and we built upon its foundation using a modified e-Delphi. This resulted in a set of 46 items that we categorised using the alphabet acronym. By working closely with the Dutch organisation with a special interest in respiratory medicine (CAHAG), we were able to embed our new programme ‘The A–I of difficult-to-manage asthma’ within existing structures of education, facilitating its implementation. Our systematic approach can be used by other healthcare systems, so each country can create its own locally adapted version of the SIMPLES difficult-to-manage asthma programme.

### Interpretation of findings in relation to previously published work

Our programme is based on the UK-based SIMPLES approach by Ryan *et al.*^16^ However, instead of a direct translation of this acronym, we decided to use it as a source of items for the Delphi process, alongside the opinion of several experts. The advantage of this approach is that it incorporates views from local experts, who know the Dutch situation and additionally it creates commitment to the programme, as they were involved in the design. In Supplementary Table 7 of the online supplement, we provide a comparison between the original SIMPLES acronym and our A–I of difficult-to-manage asthma. All original elements of the SIMPLES approach are represented in our programme. In addition, our alphabet starts with establishing the diagnosis. ‘A’ stands for: Is it asthma, what type of asthma and is it only asthma*?* A correct diagnosis and understanding of phenotypes is a logical start to the assessment of a difficult-to-manage asthma patient, especially in primary care where there are concerns about overdiagnosis, which is estimated to be present in a significant proportion of patients registered as having asthma.^25,26^ Another addition is the specific mentioning of exacerbations (E), whereas this is incorporated as part of Monitoring in SIMPLES. Bel *et al.*^14^ provide an overview of severe and difficult-to-manage asthma, and they advise the assessment of the following: persistently poor compliance (addressed in 'Device'), persistent environmental exposure to allergens or toxic substances ('Bronchial triggers'), psychosocial factors ('General behaviour'), dysfunctional breathing, vocal cord dysfunction and untreated or undertreated comorbidities such as chronic rhinosinusitis, reflux disease or obstructive sleep apnoea ('is it asthma, is it only asthma'). As shown, all of these are represented in our programme. An additional advantage of following the alphabet is that it provides an extensive personalised review of potentially treatable factors, building up to an individual care plan (the letter I) that incorporates all the treatable traits of a particular patient and assigns goals to them. A similar approach with a Delphi procedure was recently used in the CORONA study, which successfully implemented an asthma and chronic obstructive pulmonary disease (COPD) diagnostic programme, also involving primary and hospital care.^27^

In several subgroup analyses, we assessed whether there were differences between primary and hospital care and between patients and professionals (presented in Supplementary Tables 5 and 6 of the online supplement). Generally they tended to agree on which items are important, although hospital care assigned significantly higher scores to 14 items than primary care and there were some differences regarding prioritisation. However, the content of these 14 items and differences in prioritisation do not seem to be related to a specific subject. Interestingly, patients assigned a much lower importance to assessment of current asthma control and to distinguishing phenotypes of asthma, whereas they considered recognition and acceptance of personal limitations more important. They also considered the use of a questionnaire on asthma-related quality of life (AQLQ) and a Dutch questionnaire on symptom severity (RIC-MON 10) more important than professionals. However, it is important to note that the total amount of participants for these subgroups were quite small, especially for the patient group (*n*=6), so we should be cautious when interpreting these results. Also, in comparison to the overall group, patients were somewhat younger (all below 50 years old) and more (83%) were female.

### Strengths and limitations of this study

A strength of our study is that by using a Delphi approach we were able to gather a wide range of ideas. By involving future trainers and users of the programme in its creation, it engenders commitment. Furthermore, by involving professionals from primary and hospital care and also patients, we ensured the opinions of all relevant people in the management of asthma were included. We adopted a holistic approach to the management of difficult-to-manage asthma and by emphasising the importance of self-management and an individualised care plan, we support patients to adapt and self-manage in the face of social, physical and emotional challenges, which is proposed as the new definition of health.^28^ Promoting self-management is pivotal in promoting personalised medicine, thus optimising individual medication prescription and all other behavioural aspects.

A key part of our study was that we ensured our final programme was adapted to the local situation. Each country has a different healthcare system. In the Netherlands, asthma is predominantly managed in primary care, mainly by practice nurses (PNs) under the guidance of general practitioners (GPs). Furthermore, there is significant interest in asthma by policymakers, because asthma is seen as one of the chronic non-communicable diseases in which management could increasingly be shifted from hospital to primary care. The local situation might be very different in another country, so a proper analysis of reimbursement and other policy-related context should be an integral part of an implementation programme in other countries. Furthermore, this approach ensures that the correct patient is treated in the right place, with referral to hospital care only after adequate assessment in primary care.

In addition, by working closely with the Dutch organisation for GPs and PNs with a special interest in asthma and COPD (CAHAG), we ensured ownership by key stakeholders, facilitating implementation. Members of the CAHAG, specialised in developing educational training programmes for courses will be asked to write an educational programme based on this ‘alphabet’ approach. ‘The A–I of difficult-to-manage asthma’ will be taught regionally to GPs and PNs, by GPs and PNs with a special interest in asthma and COPD. In addition, it will be taught as a 2 h workshop on the ‘Adembenemend’ (‘Breath-taking’) course, which is a 2-day course on the management of asthma and COPD in primary care for care teams consisting of GPs and their PNs.

Our study has several limitations. First of all, we have used a modified version of the Delphi procedure, with a cut-off of 80% scoring 5, 6 or 7 on a seven-point Likert scale. Although this is a frequently used cut-off, it is arbitrary and cut-offs vary widely between different Delphi exercises.^29,30^ Also, using the 80% cut-off criterium instead of looking at mean scores resulted in some items being selected although their mean scores were lower than other items that were not selected (see Table 2). However, this criterium ensured that an item was deemed important by the majority of our experts, whereas a mean score could have been significantly influenced by a minority feeling very strongly in favour (or against) a specific item. In addition, we did not give a feedback for the previous round median scores as is done in classic Delphi processes. This was to enable categorisation of items between rounds two and three, which meant that the prioritisation was within the group rather than across all the items. Although this did not permit a formal consensus to be achieved, it facilitated the development of structured educational categories, which in turn will facilitate implementation.

Another limitation is that we did not address why specific items were chosen or deleted. Our focus was on difficult-to-manage asthma, neither asthma management in general nor specific treatment for severe asthma. This may have affected participant responses. Therefore, some low-priority scores may reflect a perception that an item was not important generally in asthma management, whereas other low-priority scores may only be perceived as not important in the context of difficult-to-manage asthma. Finally, GPs and PNs without a special interest in asthma were not selected for our Delphi, and the results could have been different if we had. However, as the topic of this programme, difficult-to-manage asthma as opposed to severe asthma, is relatively new for most GPs, we decided to only include GPs with special expertise on the subject.

### Implications for future research, policy and practice

Asthma is one of the chronic non-communicable diseases whose management could be shifted more from hospital to primary care. The ‘A–I of difficult to manage asthma’ programme facilitates a proper allocation of patients with more severe asthma to hospital care, by selecting and treating the difficult-to-manage aspects earlier in primary care. Although our algorithm is not designed to aid in the treatment of severe asthma, the algorithm still draws attention to the different phenotypes of severe asthma by questioning ‘The role of different phenotypes of asthma’ in category A (Is it asthma, what type of asthma and is it only asthma?). In addition, patients who do not reponse to the A–I approach, might be identified as having severe asthma who require referral to secondary care for targeted therapy. Although several aspects of difficult-to-manage asthma can be therapy resistant and still require hospital care, implementation of the programme has the potential to reduce the total number of referred patients. In addition, it will allow earlier referral back to primary care after hospital assessment. This might be of interest not only for patients and workers in healthcare but also for policymakers and health insurance companies. The impact on primary care management and healthcare outcomes, however, requires assessment in well-designed implementation studies. Future research should encompass implementation outcomes (such as knowledge gained and change in practice by GPs who followed the programme), as well as patient health outcomes (such as hospitalisations and measures of control) and a cost-effectiveness analysis, to determine the success of implementing our programme.

In addition, our results show that both among patients and professionals, there is considerable emphasis on patient-specific and behavioural issues, suggesting that these were seen as particularly important in people with difficult-to-manage asthma. Future research and management should be designed taking into account this particular emphasis.

### Conclusions

In this study, we present the use of a modified e-Delphi to adapt a UK-designed approach to managing difficult-to-manage asthma for implementation in the Netherlands. The ‘A–I of difficult-to-manage asthma’ programme is a robust and holistic approach to the primary care management of difficult-to-manage asthma, based on the opinions of all relevant stakeholders. The inclusive process engaged key people and organisations in the Netherlands such that the programme has been adopted and will be implemented as a routine component of training.

## Materials and methods

### Participants

We defined a relevant subset of expert and stakeholder groups for a difficult-to-manage asthma approach. This included expert GPs and PNs from the Dutch organisation for GPs with a special interest in asthma and COPD (CAHAG). In addition, we approached expert pulmonary physicians of the Working group Allergy and Asthma from the Dutch society of pulmonologists (NVALT) and respiratory nurses working in hospital care recommended by their professional society (V&VN). Finally, we approached patients, who are members of the asthma patient forum of the Lung Foundation Netherlands. All of these special interest groups have members from all over the Netherlands, from different practices and hospitals.

### Study design

We used a modified e-Delphi approach with three rounds^31–34^ to tailor the SIMPLES approach to the Dutch situation.

Round 1. We aimed to obtain all potentially relevant items for a programme about difficult-to-manage asthma. Therefore, we performed a literature review for potential items ^4,14–16,35–37^ and included items from the SIMPLES approach. In addition, we selected GPs and PNs with a special interest in asthma and respiratory nurses, asking them to suggest additional items for a difficult-to-manage asthma programme. Experts were invited by an e-mail with a short explanation about the background of the study. In addition, we presented a workshop at a national meeting of GPs with a special interest in asthma and asked all the delegates to provide us with a selection of important items for each of three categories: ‘Diagnosis and Monitoring’, ‘Pharmacological therapy’ and ‘Non-pharmacological management’.

Round 2. At this stage, the pulmonary physicians, respiratory nurses and people with asthma were added to obtain input from all relevant experts and stakeholders and to validate and rate the findings of round 1. All the participants were sent an e-mail with a link to a web-based questionnaire (using Survey Monkey) and an invitation letter, explaining the background and relevance of the study. A reminder was sent after 3 weeks if we had received no response. The online questionnaire included a list of all potential items from the first round, and the respondents were asked to rate the clinical importance of each item on a seven-point Likert scale (1=completely irrelevant, 7=highly relevant). An item was selected as relevant if 80% or more of the participants indicated the item as of above-average importance (i.e., a score of 5, 6 or 7). This analysis and further exploratory analyses were performed with STATA 11.0 (StataCorp, College Station, TX, USA).

Round 3. In this round, all the selected items from the second round were grouped into categories, and respondents from round 2 were asked to rank the items according to relevance within each category.

Finally, the items within each category were re-classified within an educational approach that resonated with existing educational strategies in the Netherlands and a new acronym was designed. The ranking allowed for a distinction between more and less relevant items, which could be used to prioritise items in time-limited training programmes.

### Subgroup analyses

In two exploratory analyses, we assessed whether there were substantial differences between patients and professionals (online supplement, Supplementary Table 5a) and between primary care and hospital care (online supplement, Supplementary Table 5b). A substantial difference between two groups was arbitrarily defined as a difference of 0.7 points or more on the mean outcome (i.e., a difference of ⩾10%). Furthermore, we assessed whether a particular group was more inclined to prioritise specific items, by calculating odds ratios. Finally, we analysed whether the results of the prioritisation of items would have differed when solely using the results from primary or hospital care (online supplement, Supplementary Table 6).

## Figures and Tables

**Table 1:** Baseline characteristics

*Characteristic*	N *(%)*
*Background*	
General practitioner	20 (45%)
Practice nurse	6 (14%)
Pulmonologist	8 (18%)
Respiratory nurse	4 (9%)
Asthma patient	6 (14%)

Gender % female	26 (59%)
	
*Age*	
18–30 y	1 (2%)
31–40 y	8 (18%)
41–50 y	15 (34%)
51–65 y	18 (41%)
65+ y	2 (5%)

**Table 2:** Overview of items selected in round 1 and the results of the Delphi procedure

*Name item*	*Percentage of participants that scored ⩾5*	*Mean score*	*Selected*^a^
*Diagnosis and monitoring*			
Explanation what is difficult-to-manage asthma	98%	6.3	Yes
Identification of patients with difficult-to-manage asthma	93%	6.1	Yes
What is the difference between difficult-to-manage asthma and severe asthma	90%	5.9	Yes
Further insight into the potential consequences of a diagnosis of severe asthma (such as the use of biologicals, revalidation-therapy, high-altitude treatment.)	88%	5.7	Yes
Has asthma been diagnosed according to the current guidelines	81%	5.8	Yes
The role of comorbid diseases in asthma	76%	5.4	No
Differential diagnosis of asthma and which potential concurrent diagnoses require further investigation/management	86%	5.6	Yes
Which other tests should/could be performed other than symptoms and spirometry, to diagnose asthma	86%	5.3	Yes
Role of blood eosinophils	62%	4.9	No
Role of NT-BNP	36%	4.1	No
Role of histamine-provocation	71%	5.1	No
Role of FeNO	45%	4.5	No
Role of pulmonary imagining	40%	4.1	No
Role of the ACQ	83%	5.6	Yes
Role of the AQLQ	48%	4.6	No
Role of the RIC-MON 10	26%	3.7	No
Role of spirometry	71%	5.3	No
Role of different phenotypes of asthma	83%	5.7	Yes
How to assess different phenotypes of asthma in a patient	86%	5.6	Yes
Identification of patients with an increased risk of asthma exacerbations	90%	5.8	Yes
Frequency of monitoring for the individual patient	83%	5.4	Yes
Clear guidelines for referrals between primary and hospital care	86%	5.8	Yes
Determining current control	88%	5.8	Yes
Content of monitoring in the individual patient	90%	5.8	Yes
Additional use of spirometry in patients with no symptoms and persistent obstruction	79%	5.3	No
Additional use of spirometry in patients with a lot of symptoms and a normal lung function	79%	5.3	No
Role of peak flow in daily monitoring	40%	4.0	No
Identification of patients suitable for pulmonary rehabilitation	83%	5.5	Yes
Determining general goals of treatment	74%	5.1	No
Determining personal goals of treatment.	98%	6.0	Yes
			
*Medication(-related) therapy*			
Denominate central role-inhaled corticosteroids	90%	5.9	Yes
Name common side-effects of different types of medication	93%	5.5	Yes
Pharmacotherapy for specific subgroups: does comorbidity determine medication choices	86%	5.4	Yes
Identification of suitable patients for LTRA	76%	4.9	No
Role of inhalation instruction	90%	6.2	Yes
Role of device type	88%	5.8	Yes
Inventarisation of adherence	93%	6.0	Yes
Role of particle size	62%	4.9	No
Patient perceptions on benefits and necessity of medication	93%	6.0	Yes
			
*Non-pharmacological management*			
Follow-up of comorbidity	74%	5.0	No
An asthma action plan for every asthma patient	71%	5.1	No
An asthma action plan for every difficult-to-manage asthma patient	98%	6.2	Yes
Recognition of causing agents of exacerbations	86%	5.7	Yes
Effect of smoking on asthma	90%	6.0	Yes
Role of passive smoking	86%	5.6	Yes
Other types of drugs	69%	5.1	No
Insight into aspecific irritants	95%	6.0	Yes
Insight into allergens	97%	6.0	Yes
Insight into occupational irritants	93%	5.8	Yes
Insight into hobby-related irritants	83%	5.5	Yes
Attention for physical activity	90%	5.8	Yes
Role of weight	90%	5.8	Yes
Identification of obstacles for adherence (social, financial, societal)	88%	5.7	Yes
Recognition of stress-inducing factors	81%	5.5	Yes
Self-management for all people with (difficult to manage) asthma	95%	6.0	Yes
Identification of patients suited to different types of self-management: paper, online, real-life	83%	5.5	Yes
Role of eHealth	64%	5.0	No
How to make patients aware of asthma worsening events/behaviour	93%	5.7	Yes
Education on asthma	86%	5.6	Yes
Insight into the patients personal environment	90%	5.5	Yes
Getting a patients’ environment involved in treatment	79%	5.3	No
Knowledge about the relation between symptoms and work	88%	5.6	Yes
Necessity of an individual care plan for all patients with (difficult to manage) asthma	83%	5.7	Yes
Recognition and acceptance of personal limitations	88%	5.7	Yes
Estimation of desire and potential for behavioural changes	88%	5.6	Yes

Abbreviations: ACQ, asthma control questionnaire; AQLQ, asthma quality of life questionnaire; FeNO, fractional exhaled nitric oxide; LTRA, leucotriene receptor antagonist; NT-BNP, N-terminal brain natriuretic peptide; RIC-MON 10, Dutch questionnaire on symptom severity.

a Yes if ⩾80% of participants scored ⩾5 on this item.

**Table 3:** Overview of results Delphi round 3

*Category*	*Name item*	*Rank*
Background	Explanation what is difficult-to-manage asthma	1
	Identification of patients with difficult-to-manage asthma	2
	What is the difference between difficult-to-manage asthma and severe asthma	3
	Clear guidelines for referrals between primary and hospital care	4
	Further insight into the potential consequences of a diagnosis of severe asthma (such as the use of biologicals, revalidation-therapy, high-altitude treatment.)	5
		
Diagnosis	Has asthma been diagnosed according to the guidelines	1	
	Differential diagnosis of asthma and which potential concurrent diagnoses require further investigation	2	
	The role of comorbidity in asthma	3	
	How to assess different phenotypes of asthma in a patient	3	
	Role of the ACQ	3	
	Role of different phenotypes of asthma	6	
	Which other tests should/could be performed other than symptoms and spirometry, to diagnose asthma	7	
		
Monitoring	Content of monitoring in the individual patient	1	
	Determining current control	2	
	Frequency of monitoring for the individual patient	3	
		
Exacerbation	An asthma action plan for every difficult-to-manage asthma patient	1	
	Identification of patients with an increased risk	2	
	Definition of (severe) asthma exacerbation	3	
	Recognition of causing agents of exacerbations	4	
		
Types of medication	Denominate central role-inhaled corticosteroids	1	
	Pharmacotherapy for specific subgroups: does comorbidity determine medication choices	2	
	Name common side-effects different types of medication	3	
		
Use of medication	Role of inhalation instruction	1	
	Assessment of adherence	2	
	Role of device type	3	
		
Smoking	Effect of smoking on asthma	1	
	Role of passive smoking	2	
		
Other irritants	Insight into allergens	1	
	Insight into nonspecific irritants	2	
	Insight into occupational irritants	3	
	Insight into hobby-related irritants	4	
		
Lifestyle	Identification of obstacles for adherence (social, financial, societal)	1	
	Attention for physical activity	2	
	Role of weight	3	
	Identification of patients suitable for pulmonary rehabilitation	4	
	Recognition of stress-inducing factors	5	
		
Education and self-management	Self-management for all people with (difficult-to-manage) asthma	1	
	Patient perceptions on benefits and necessity medications	2	
	Education on asthma	3	
	Identification of patients suited to different types of self-management: paper, online, real-life	4	
	How to make patients aware of asthma worsening events/behaviour	5	
		
Patient Profile	Insight into the patients' personal environment	1	
	Knowledge about the relation between symptoms and work	2	
		
Individual care plan	Necessity of an individual care plan for all patients with (difficult-to-manage) asthma	1	
	Recognition and acceptance of personal limitations	2	
	Determining personal goals of treatment.	2	
	Estimation of desire and potential for behavioural changes	4	

Abbreviation: ACQ, asthma control questionnaire.

**Table 4:** The alphabet

A	Asthma (Dutch: astma). Is it asthma, what type of asthma and is it only asthma?
	Items:
	Has asthma been diagnosed according to the guidelines Explanation what is difficult-to-manage asthma Identification of patients with difficult-to-manage asthma What is the difference between difficult-to-manage asthma and severe asthma Role of different phenotypes of asthma Which other tests should/could be performed other than symptoms and spirometry, to diagnose asthma The role of comorbidity in asthma *How to assess different phenotypes of asthma in a patient* *Differential diagnosis of asthma and which potential concurrent diagnoses require further investigation* *Further insight into the potential consequences of a diagnosis of severe asthma (such as the use of biologicals, revalidation-therapy, high-altitude treatment..)*

B	Bronchial triggers (Blootstelling). Allergens and irritants causing symptoms
	Items:
	Effect of smoking on asthma Insight into allergens Insight into nonspecific irritants *Role of passive smoking* *Insight into occupational irritants* *Insight into hobby-related irritants* *Knowledge about the relation between symptoms and work*

C	Asthma control (Controle). How to assess and monitor asthma control
	Items:
	Determining current control: ACQ, exacerbation rate, persistent obstruction Content of monitoring in the individual patient Role of the ACQ *Frequency of monitoring for the individual patient* *Clear guidelines for referrals between primary and hospital care* *Identification of patients suitable for pulmonary rehabilitation*
	
D	Device (Device). Which device and how to use it?
	Items:
	Role of inhalation instruction Assessment of adherence *Role of device type: match between patient and device*

E	Exacerbations (Exacerbaties). How to prevent, detect and treat exacerbations
	Items:
	An asthma action plan for every difficult-to-manage asthma patient Identification of patients with an increased risk *Definition of a (severe) asthma exacerbation* *Recognition of causing agents of exacerbations*

F	Pharmacotherapy (Farmacotherapie). Which types of medication for which individual patient
	Items:
	Denominate central role-inhaled corticosteroids Patient perceptions on benefits and necessity of medication Pharmacotherapy for specific subgroups: does comorbidity determine medication choices *Common side-effects different types of medication*

G	General behaviour (Gedrag). How does behaviour and lifestyle influence asthma and how to modify it
	Items:
	Attention for physical activity Role of weight *Recognition of stress-inducing factors*

H	Help (Hulp). Strengthen the knowledge and determine who can aid a patient in disease management
	Items:
	Identification of obstacles for adherence (social, financial, societal) Education on asthma Insight into the patients' personal environment

I	Individualised care plan (Individueel Zorg Plan). How to create and use a self-management plan for each individual patient
	Items:
	Self-management for all people with (difficult-to-manage) asthma Necessity of an individual care plan for all patients with (difficult-to-manage) asthma Recognition and acceptance of personal limitations Determining personal goals of treatment. *Identification of patients suited to different types of self-management: paper, online, real-life* *How to make patients aware of asthma worsening events/behaviour* *Estimation of desire and potential for behavioural changes*

Abbreviation: ACQ, asthma control questionnaire.

Items in italic were deemed less relevant in round 3 of the modified Delphi procedure.
