# Supplementary material for: Adaptation of a difficult-to-manage asthma programme for implementation in the Dutch context: a modified e-Delphi
Source: NPJ Prim Care Respir Med. 2017 Feb 9;27:16086–. doi: 10.1038/npjpcrm.2016.86 (PMC5301160; doi:10.1038/npjpcrm.2016.86)
Supplement: Supplementary Information [file npjpcrm201686-s1.doc]

ONLINE SUPPLEMENT

Table 5a. Differences between patients and professionals

| Patients assigned a substantially higher score on the following items* | Mean score professionals | Mean score patients |
| --- | --- | --- |
| What is the difference between difficult to manage asthma and severe asthma | 5.8 | 6,7 |
| Role of the AQLQ | 4.4 | 5.3 |
| Role of the RIQ-MON 10 | 3.5 | 4.8 |
| Further differentiation for specific subgroups (e.g. mentally or physically challenged) | 4.3 | 5.2 |
| Role of peak flow in daily monitoring | 3.9 | 4.8 |
| Role of passive smoking | 5.5 | 6.3 |
| Recognition of stress-inducing factors | 5.3 | 6.5 |
| Recognition and acceptance of personal limitations | 5.6 | 6.7 |
| Professionals assigned a substantially higher score on the following items* |  |  |
| Role of different fenotypes of asthma | 5.9 | 4.5 |
| Determining current control | 6 | 4.5 |

* Defined as a difference of ≥ 0.7

Table 5b Differences between primary and hospital care

| Hospital care assigned a substantially  higher score on the following items* | Mean score hospital | Mean score primary care |
| --- | --- | --- |
| The role of comorbidity in asthma | 6.0 | 5.1 |
| Role of blood eosinophils | 5.3 | 4.6 |
| Role of different phenotypes of asthma | 6.5 | 5.5 |
| Role of the AQLQ | 5.2 | 4.1 |
| Determining current control | 6.5 | 5.8 |
| Role of inhalation instruction | 6.9 | 5.9 |
| Inventarisation of adherence | 6.6 | 5.9 |
| Insight into occupational irritants | 6.3 | 5.6 |
| Attention for physical activity | 6.3 | 5.4 |
| Role of weight | 6.4 | 5.5 |
| Recognition of stress-inducing factors | 5.8 | 5.0 |
| Education on asthma | 6.2 | 5.4 |
| Getting a patients’ environment involved in treatment | 5.8 | 4.9 |
| Knowledge about the relation between symptoms and work | 6.0 | 5.3 |

* Defined as a difference of ≥0.7

Table 6 Differences between overall choices of most relevant items in the alphabet and results from primary and hospital care

| Category | Name item * | Primary care Rank † | Hospital care rank ‡ |
| --- | --- | --- | --- |
| Background | Explanation what is difficult to manage asthma | 3 | 1 |
|  | Identification of patients with difficult to manage asthma | 1 | 2 |
|  | What is the difference between difficult to manage asthma and severe asthma | 2 | 3 |
|  | *Clear guidelines for referrals between primary and hospital care* | 4 | 4 |
|  | *Further insight into the potential consequences of a diagnosis of severe asthma (such as the use of biologicals, revalidation-therapy, high-altitude treatment..)* | 5 | 5 |
| Diagnosis | Has asthma been diagnosed according to the guidelines | 2 | 2 |
|  | Differential diagnosis of asthma and which potential concurrent diagnoses require further investigation | 1 | 4 |
|  | The role of comorbidity in asthma | 3 | 5 |
|  | How to assess different phenotypes of asthma in a patient | 5 | 3 |
|  | Role of the ACQ | 6 | 1 |
|  | *Role of different phenotypes of asthma* | 4 | 6 |
|  | *Which other tests should/could be performed other than symptoms and spirometry, to diagnose asthma* | 7 | 7 |
| Monitoring | Content of monitoring in the individual patient | 2 | 2 |
|  | Determining current control | 1 | 1 |
|  | *Frequency of monitoring for the individual patient* | 3 | 3 |
| Exacerbation | An asthma action plan for every difficult to manage asthma patient | 1 | 1 |
|  | Identification of patients with an increased risk | 2 | 2 |
|  | *Definition of a (severe) asthma exacerbation* | 3 | 3 |
|  | *Recognition of causing agents of exacerbations* | 4 | 4 |
| Types of medication | Denominate central role inhaled corticosteroids | 1 | 1 |
|  | Pharmacotherapy for specific subgroups: does comorbidity determine medication choices | 2 | 2 |
|  | *Name common side-effects different types of medication* | 3 | 3 |
| Use of medication | Role of inhalation instruction | 1 | 1 |
|  | Assessment of adherence | 2 | 2 |
|  | *Role of device type* | 3 | 3 |
| Smoking | Effect of smoking on asthma | 1 | 1 |
|  | *Role of passive smoking* | 2 | 2 |
| Other irritants | Insight into allergens | 1 | 1 |
|  | Insight into non-specific irritants | 2 | 2 |
|  | *Insight into occupational irritants* | 3 | 3 |
|  | *Insight into hobby-related irritants* | 4 | 4 |
| Lifestyle | Identification of obstacles for adherence (social, financial, societal) | 4 | 1 |
|  | Attention for physical activity | 1 | 2 |
|  | Role of weight | 2 | 4 |
|  | *Identification of patients suitable for pulmonary rehabilitation* | 3 | 3 |
|  | *Recognition of stress-inducing factors* | 5 | 5 |
| Education and self-management | Self-management for all people with (difficult to manage) asthma | 2 | 1 |
|  | Patient perceptions on benefits and necessity medications | 4 | 2 |
|  | Education on asthma | 1 | 4 |
|  | *Identification of patients suited to different types of self- management: paper, online, real-life* | 3 | 5 |
|  | *How to make patients aware of asthma worsening events/behaviour* | 5 | 3 |
| Patient Profile | Insight into the patients personal environment | 1 | 2 |
|  | *Knowledge about the relation between symptoms and work* | 2 | 1 |
| Individual care plan | Necessity of an individual care plan for all patients with (difficult to manage) asthma | 1 | 1 |
|  | Recognition and acceptance of personal limitations | 2 | 3 |
|  | Determining personal goals of treatment. | 3 | 2 |
|  | *Estimation of desire and potential for behavioural changes* | 4 | 4 |

* Items in *italic* were deemed less relevant in the current version of the alphabet (according to overall results in round 3 of the modified Delphi procedure).

† Relevance of items when solely responses of primary care professionals are taken into account. Highlighted in green when it would have been selected and in red if not

‡ Relevance of items when solely responses of hospital care professionals are taken into account. Highlighted in green when it would have been selected and in red if not

Table 7. Comparison between original SIMPLES and A-I of difficult to manage asthma

| SIMPLES | A-I program |
| --- | --- |
| Smoking | B. Bronchial Triggers |
| Inhaler technique | D. Device |
| Monitoring | C. Asthma Control |
| Pharmacotherapie | F. Pharmacotherapy |
| Lifestyle | G. General behaviour |
| Education | H. Help |
| Support | I. Individual care plan (and H Help) |
| * | A. Is it Asthma |
|  | E. Exacerbations |

* Not represented in SIMPLES
